# An Atlas of Piezoelectric Energy Harvesters in Oceanic Applications

**DOI:** 10.3390/s22051949

**Published:** 2022-03-02

**Authors:** Seyyed Masoud Kargar, Guangbo Hao

**Affiliations:** School of Engineering and Architecture, University College Cork, T12K8AF Cork, Ireland; 120222847@umail.ucc.ie

**Keywords:** piezoelectric, energy harvesting, ocean, energy conversion, atlas

## Abstract

Nowadays, a large number of sensors are employed in the oceans to collect data for further analysis, which leads to a large number of demands for battery elimination in electronics due to the size reduction, environmental issues, and its laborious, pricy, and time-consuming recharge or replacement. Numerous methods for direct energy harvesting have been developed to power these low-power consumption sensors. Among all the developed harvesters, piezoelectric energy harvesters offer the most promise for eliminating batteries from future devices. These devices do not require maintenance, and they have compact and simple structures that can be attached to low-power devices to directly generate high-density power. In the present study, an atlas of 85 designs of piezoelectric energy harvesters in oceanic applications that have recently been reported in the state-of-the-art is provided. The atlas categorizes these designs based on their configurations, including cantilever beam, diaphragm, stacked, and cymbal configurations, and provides insightful information on their material, coupling modes, location, and power range. A set of unified schematics are drawn to show their working principles in this atlas. Moreover, all the concepts in the atlas are critically discussed in the body of this review. Different aspects of oceanic piezoelectric energy harvesters are also discussed in detail to address the challenges in the field and identify the research gaps.

## 1. Introduction

With the development of oceanic industries, the risks of polluting the oceans are gradually increasing. To solve this type of problem, ocean monitoring devices, such as different kinds of sensors, are developed. Sensors can collect data from distant places and send it to the stations nearby [1].

Employing technologies, such as Micro-Electro-Mechanical-Systems (MEMS), has introduced many low-energy consumption sensors to ocean pollution and water quality analyses. Miniaturization, diminishing power consumption, and portability are the most common trends in the newly emerged sensors and electronic devices [2]. These sensors simply collect data from the ocean and send it to stations for further analysis. Conventionally, these devices have been powered by chemical batteries. However, most often, the life of the batteries is shorter than the life of the sensors. Therefore, the batteries require replacement or recharge, and both of these actions are very pricy, time-consuming, and laborious. Moreover, as batteries are bulky and heavy, they hinder the development of miniature, as well as light, electronic devices. On the other hand, the possibility of polluting the environment by the leaking of the dangerous chemicals inside batteries threatens oceans and the animals living in them. Therefore, it is of high importance to eliminate batteries from oceanic devices and make them self-powered [3,4].

Considering that there are many types of renewable energy sources in the oceans in the form of mechanical energy (such as waves, tides, and currents), a high amount of research is devoted to developing technologies for energy conversion. Most recently, as oceans contain a huge amount of energy and are completely predictable and reliable, the generation of electricity based on oceans is becoming very popular. Electricity generation from oceanic energies can be either on large-scales [5], such as wave activated bodies [6], point absorbers [7,8], oscillating water columns [9], and overtopping [10] concepts, for cities and industries, or on small-scales using piezoelectric [11], electrostatics [12], electromagnetics [13], and triboelectric [14] devices for powering low power devices, such as the internet of underwater things, sensors, and other monitoring devices in oceans [15].

Recent progress in oceanic energy harvesting shows that batteries and cables will be eliminated in future electronics and will be replaced by clean power that does not introduce any environmental issues.

Among different small-scale energy harvesters, piezoelectric materials show a considerable energy generation density that is about three times higher than the others [16]. Moreover, these materials can be simply attached to the systems, and as they contain no moving parts, they are free from frequent upkeep. Moreover, they have the merits of direct conversion of energy to electricity, being compact, structurally simple, clean, light-weight, stable, and very sensitive to any small strains. Although the generated electricity by piezoelectric energy harvesters is rather small, it is enough for low energy consumption electronics, such as oceanic sensors [3].

Numerous piezoelectric energy harvesters with suitable properties for different applications have been developed. These include applications in transportation [17], smart systems [18], microfluidics [19], tissue engineering [20], implantable/wearable electronics [21], biomedical engineering [22], wind energy [23], and ocean energy [24]. Piezoelectric materials started to be used in the ocean as a power extractor mechanism in the 1970s [11]. However, large-scale (kW range) power generation from ocean energy making use of piezoelectric materials is still under research [25].

Considerable excellent state-of-the-art surveys have been provided in the field of piezoelectric energy harvesters and oceanic piezoelectric energy harvesters. Jbaily et al. considered different aspects of piezoelectric devices and their working mechanism in oceanic applications [11]. Viet et al. compared the other energy harvesters in oceanic applications with piezoelectric energy harvesters and categorized the former literature and prototypes based on their piezoelectric coupling modes [26]. Kim has reviewed electroactive polymers for ocean kinetic energy harvesting [27]. Kiran et al. presented a review of different aspects of piezoelectricity and provided a historical state-of-the-art in the piezoelectric energy harvesters in the ocean [28]. There are, however, other reviews in the field [2,3,29] but none of them compiled a comprehensive atlas of piezoelectric energy harvesters’ design. The present study is entirely dedicated to piezoelectric energy harvesters in oceanic applications because they are receiving massive attention most recently and growing quickly. In this regard, the next section of this review is dedicated to oceanic piezoelectric energy harvesters, which reviews piezoelectricity, piezoelectric materials in ocean energy harvesting applications, piezoelectric coupling modes, and classification of piezoelectric energy harvesters based on device structure, power harvesting system, ocean energy sources, and the location of the oceanic facilities. Next, the atlas of 85 piezoelectric energy harvesters is presented through four classifications; (1) Cantilever beam-based piezoelectric energy harvesters; (2) Diaphragm-based piezoelectric energy harvesters; (3) Stacked-based piezoelectric energy harvesters; (4) Cymbal-based piezoelectric energy harvesters. In the end, the future perspectives and challenges are discussed thoroughly.

## 2. Oceanic Piezoelectric Energy Harvesters

### 2.1. Piezoelectricity

**Figure 1:**
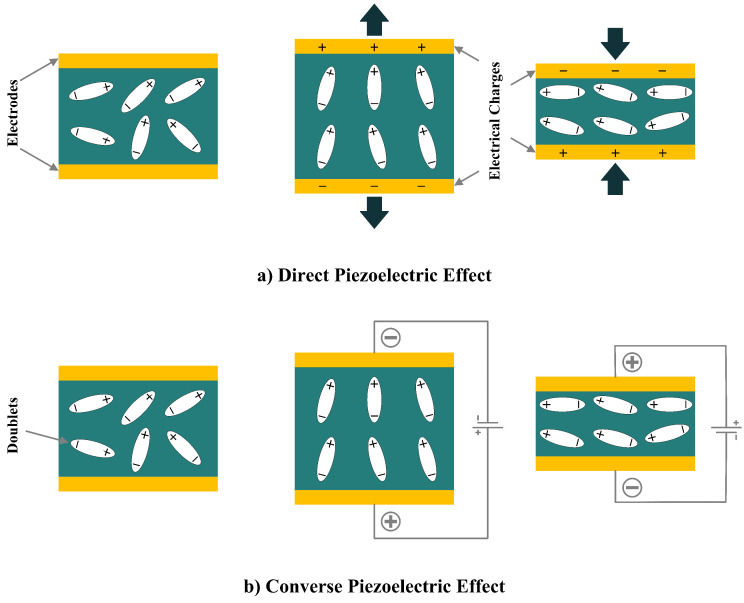
Piezoelectric Effect; (**a**) Direct Piezoelectric Effect; (**b**) Converse Piezoelectric Effect.

In 1880, the term piezoelectricity was introduced by brothers Pierre and Jacques Curie [30], which has been intensively studied in recent years for different sensory and actuation applications in different fields. Later, in 1881, the brothers Curie validated their work on the inverse piezoelectric effect with experiments [3]. Stressing a piezoelectric material would result in a change in its atomic configuration, which forms dipole moments. This phenomenon is called the direct piezoelectric effect, in which the piezoelectric material generates electricity as a reaction to the applied force. Therefore, if a periodic force (tension or compression) is applied to the material, an alternative current (AC) voltage will be the output. On the other hand, if the piezoelectric material is electrically polarized, the converse piezoelectric effect appears in which the piezoelectric material extends/contracts as a result of the applied electrical voltage. It should be noted that in the inverse piezoelectric effect, lengthening or shortening of the piezoelectric material depends on the polarity and the applied poling voltage and can be reversed if the voltage direction is reversed [11]. Both the direct and converse effects of piezoelectricity are shown in Figure 1.

There are many diverse applications for both direct and inverse piezoelectric effects, where the direct effect is utilized in the case of sensors and energy harvesters, and the inverse effect is used in actuators. These effects are governed by piezoelectric constitutive equations as [31]:

(1) D=dT+εE

(2) X=sT+dE

where *D*, *d*, *T*, ε, *E*, *X*, and *s*, represent electrical displacement, piezoelectric coefficient, stress, the permittivity of the material, electric field, strain, and mechanical compliance, respectively.

### 2.2. Piezoelectric Materials

Active materials that can generate electricity as a reaction to small mechanical stress are called piezoelectric materials. The type of material for energy harvesting applications is of high importance to the performance and functionality of the harvester. Researchers have investigated numerous types of materials, such as organic, inorganic, composite, and bio-inspired materials, for piezoelectric energy harvesters [31]. Among them, PZT and PVDF are the most widely used materials for energy harvesting in oceanic applications. PZT, which is also known as Lead Zirconate Titanate, is a polled ferroelectric ceramic with the highest frequency of applications in piezoelectric energy harvesters. Even though the PZT is one of the major materials in energy harvesting, its drawbacks, such as its drastic brittle nature and fatigue growth possibility in high-frequency loadings, limited its widespread usage in energy harvesting. On the other hand, to overcome the demerits of PZT, PVDF, also known as polyvinylidene fluoride, is developed to improve the efficiency of piezoelectric energy harvesters [32]. Although PZT can produce more electricity when subjected to the same stress as PVDF, unlike PVDF, it cannot bear high stress. PVDF shows a higher tensile strength value, which is about 2.6 times higher than PZT’s strength value [11]. The low stiffness, large tensile strength, and high flexibility of PVDF make it a unique choice for many applications, specifically in ocean energy harvesters. Apart from PZT and PVDF, macro-fiber composites (MFC) are also used in some of the literature in ocean applications, which show great performance in energy harvesting from the ocean.

### 2.3. Coupling Modes

Piezoelectric materials present a kind of flexibility in their configurations, which makes them unique in energy harvesting applications. Based on the desired configuration in a particular application, piezoelectric materials’ configurations can be altered by changing their electrode pattern, poling direction, and strain direction. Moreover, to tune the resonant frequency between the material and the energy source, volume and layers can be changed, and a pre-load force can be added [27].

**Figure 2:**
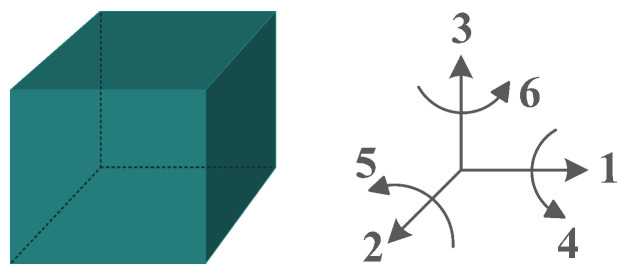
Polarization axes of piezoelectric materials.

**Figure 3:**
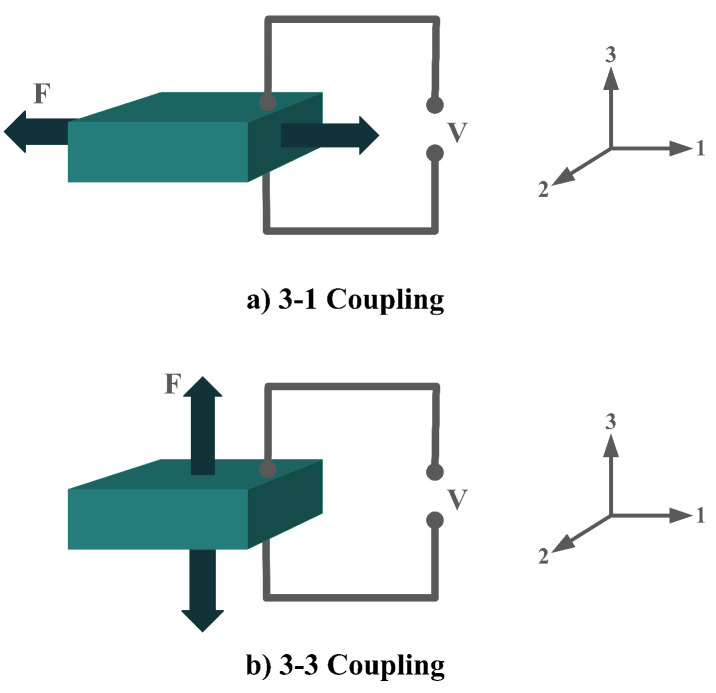
Piezoelectric Coupling Modes; (**a**) 3-1 Coupling Mode; (**b**) 3-3 Coupling Mode.

The piezoelectric coefficient (*d_ij_*) is the ratio of the strain to the electric field, where *i* denotes the direction of the polarity and *j* represents the mechanical stress direction. Figure 2 shows piezoelectric material axes for polarization, as well as stress/strain. According to [28], d_14_, d_15_, d_33_, and d_31_, or simply 1-4, 1-5, 3-3, and 3-1, respectively, are the piezoelectric energy harvesters’ configurations in oceanic applications. However, due to the complex configurations of 1-4 and 1-5 modes, they are less used in oceanic applications compared to 3-3 and 3-1 modes. Figure 3 shows the most utilized configurations, 3-3 and 3-1, in ocean applications. In the 3-3 mode, both the polarity and the mechanical stress are in the same direction, parallel to each other. However, in the case of 3-1, the direction of the mechanical stress is perpendicular to that of polarity [33]. It should also be noted that choosing the right configuration for designing a high-performance piezoelectric energy harvester plays a crucial role and thus should be carefully considered based on the design requirements.

### 2.4. Classification Based on Device Structure

**Figure 4:**
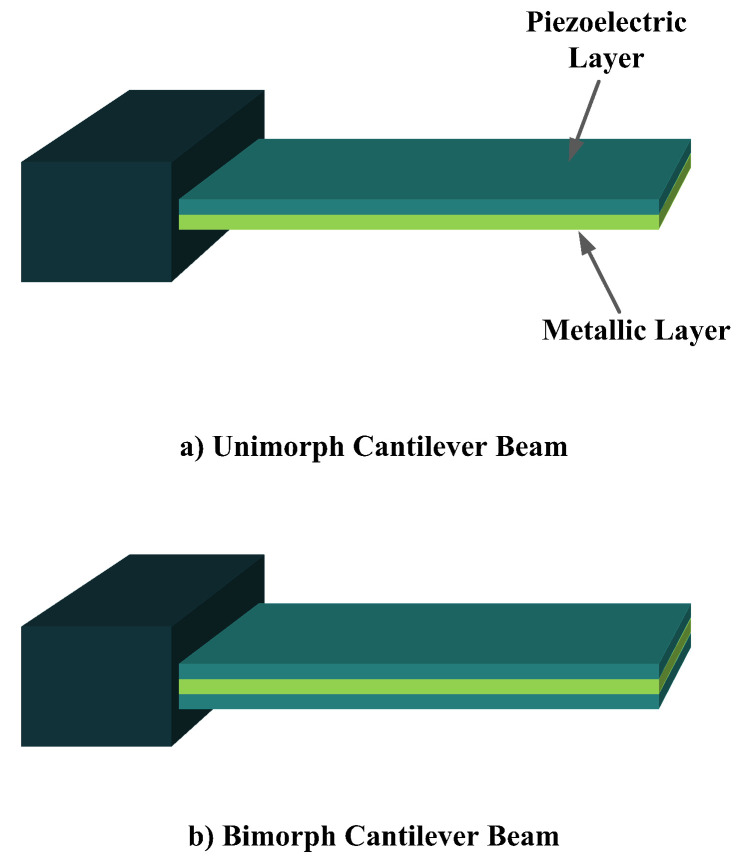
Cantilever Beam Configuration; (**a**) Unimorph; (**b**) Bimorph.

The most common structure for a piezoelectric energy harvesting device is the cantilever beam configuration. This type of harvester employs one or two piezoelectric layers and is called unimorph and bimorph, respectively. Figure 4 shows cantilever beam structures with one and two layers of piezoelectric materials. As can be seen in Figure 4, piezoelectric layers are mostly bonded to a metallic non-piezoelectric material that is fixed at one end and acts as a flexural structure. As it is clear, taking advantage of two layers of piezoelectric materials, bimorph-type cantilever beams are capable of generating more output power compared to unimorph configurations. Therefore, bimorph configurations are of the high frequency of applications in piezoelectric energy harvesters [34,35].

Cantilever beam configuration utilizes the 3-1 coupling mode most often. However, there are designs in the literature that have used 3-3 coupling modes for cantilever structures, making use of interdigitated electrode designs. Moreover, it is usual to add a proof mass at the free end of the cantilever beam to tune the resonant frequency of the harvester with the environment [31].

**Figure 5:**
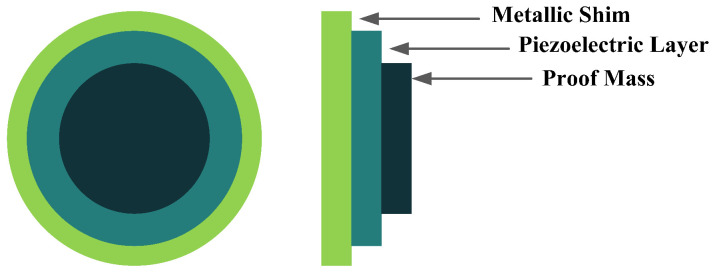
Diaphragm Configuration.

The other configuration, which consists of a piezoelectric layer with a mostly disk shape, is the diaphragm structure. The piezoelectric layer is bonded to a metal shim. In some configurations, to improve functionality under low frequencies and to add a pre-loading unit to the energy harvester, a proof mass is attached to the diaphragm’s core (Figure 5) [36].

**Figure 6:**
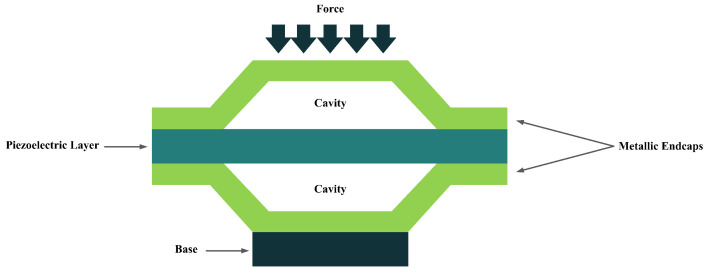
Cymbal Configuration.

Adding two metallic endcaps in the shape of a cymbal on both sides of a piezoelectric disk would result in a new configuration called the cymbal structure (Figure 6). This configuration is indeed used to improve piezoelectric endurance when it is subjected to higher loads and impact forces. Moreover, the cymbal shape of the endcaps acts as a mechanical amplification unit due to the presence of the cavity in the center [36].

**Figure 7:**
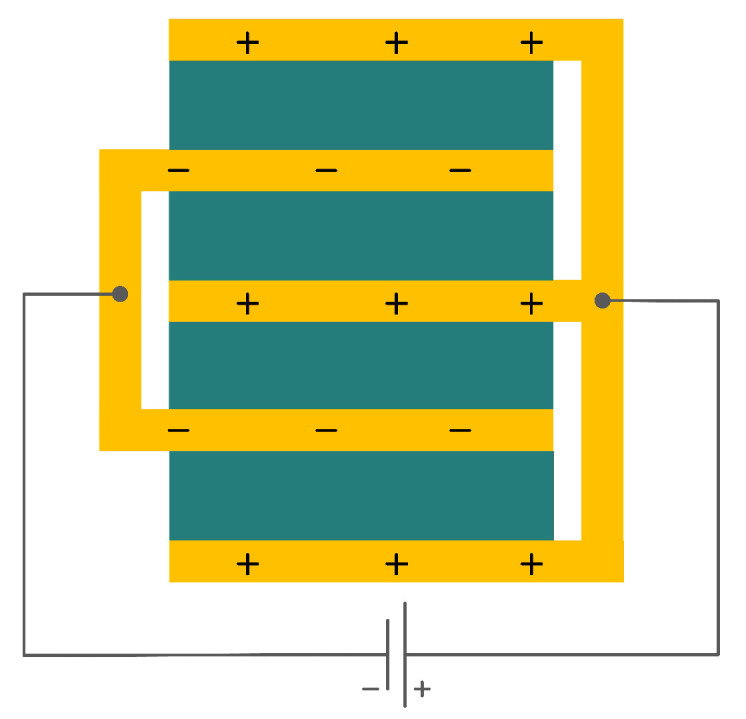
Stacked Configuration.

In addition to the other configurations, stacked piezoelectric structures can bear higher pressures. This configuration is made up of multiple layers of piezoelectric material stacked on top of each other. The poling direction of this type should be aligned with the applied force direction, as shown in Figure 7 [35].

All the aforementioned configurations have their own merits and drawbacks. Each of them may be suitable for numerous applications and unsuitable for many others. Therefore, before employing one of them in an application, their limitations should be carefully studied and considered. In addition to the cantilever beam configuration’s advantages, such as simple structure, low price, suitability for low-frequency applications, and higher mechanical quality factor, they are not able to withstand high impact forces. On the other hand, the cymbal configuration can bear impact forces and provide high energy output. However, the loss of mechanical input energy and being limited to applications requiring high vibration sources are its drawbacks. The circular diaphragm configuration is capable of working in pressure mode operations, but in a vibration mode application, it requires higher resonance frequencies. The stacked configuration is also suitable for working in pressure mode. It can also bear higher mechanical forces and provide higher outputs in the d_33_ coupling mode. Nevertheless, its high stiffness is among the challenges that should be addressed in the search for a proper application for it [36].

### 2.5. Power Harvesting System

**Figure 8:**
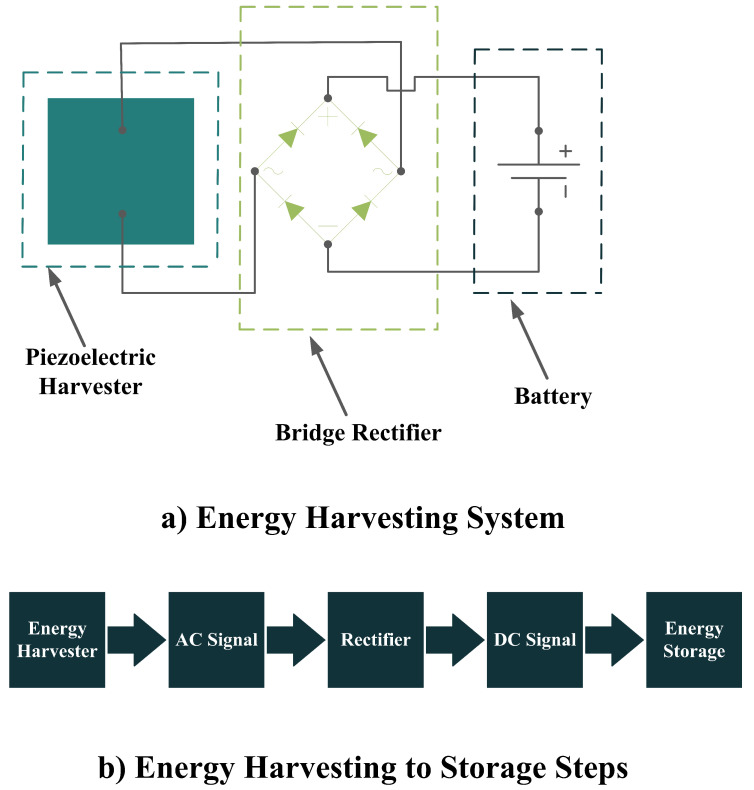
Power Harvesting Operation; (**a**) Energy Harvesting System; (**b**) Energy Harvesting to Storage Steps.

The generated electricity by the piezoelectric energy harvesters needs to be further processed before any collection or storage in a power storage system. However, it should be taken into consideration that power storage systems, such as batteries, require direct current (DC) rather than alternative current (AC). If the piezoelectric harvester acts as a resonator, then the resultant voltage would be a sinusoidal signal, which should be rectified for any further applications. To carry out this operation, an intermediate step needs to be implemented to convert AC signals to DC. To do so, either the Standard Technique or the Synchronized Switch Harvesting on Inductor Technique can be employed [37,38]. By eliminating the ripple voltage, these techniques would smooth out the DC signal, and after regulation by the control system, the signal is ready to be stored in a power storage system. Figure 8 provides an illustration of the power harvesting operation by piezoelectric materials to further simplify the understanding of the process.

**Figure 9:**
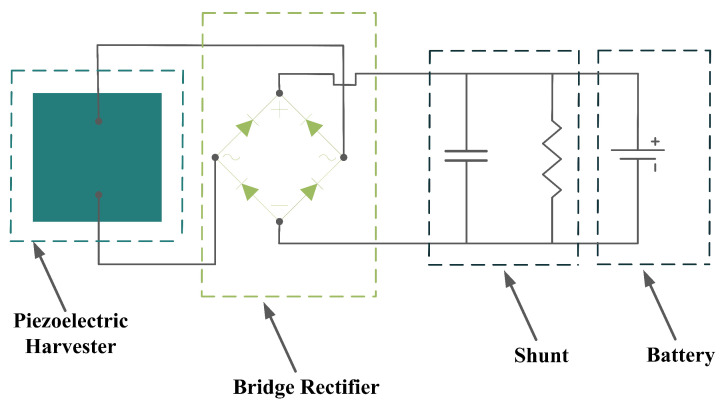
Energy Harvesting System with an Impedance Matching Unit (Shunt).

In addition to the circuit shown in Figure 8, there should be an impedance-matching system in between to guarantee the high performance of the piezoelectric energy harvesters. Matching the electrical parameters with the source, cable, or receiver is of high importance for any electrical transmission line that is involved in the transfer of an electrical signal or power. Therefore, the impedance matching of the electrical parameters should be carefully considered in the design stage of the piezoelectric energy harvesters [39]. According to [39], matching of both acoustic and electrical components in a design should be considered for impedance matching. However, in the case of piezoelectric energy harvesters, the impedance matching of electrical components is more important. Piezoelectric energy harvesters are narrow-banded intrinsically, but a broadband operation is required for energy harvesting, in which acoustic impedance matching is employed to improve the narrow-band operation. However, it increases the electrical impedance and leads to an impedance mismatch between the harvester and the interface device. This mismatch diminishes the electric energy and requires an electrical impedance matching unit to be minimized. To do so, a shunt circuit [40], which is the simplest one for this purpose, can be employed to maximize the output electrical energy (Figure 9). One can refer to [39,40,41,42] for more information regarding the impedance matching of piezoelectric energy harvesters.

### 2.6. Ocean Energy Sources for Piezoelectric Energy Harvesters

Marine renewable energies are the source of many different energy converters [5,43]. In the case of piezoelectric energy harvesters, water current, wave motion, and wave impact forces have been employed to extract energy from the ocean and convert it to usable electrical energy to power sensors and some other measurement devices [11]. These sources are very promising and available at all times during the day. Moreover, these energy sources in marine environments have a high density compared to the other sources of renewable energies, such as wind or solar energies [44]. In the following, a short introduction will be given to each of the marine energy sources that piezoelectric energy harvesters are designed for.

#### 2.6.1. Water Currents

To employ piezoelectric energy harvesters to extract power from ocean water currents, the vibration in the water can be converted to electricity. To do so, the piezoelectric energy harvester should be placed in the current stream and be coupled with flow-induced vibration. Vortex-Induced Vibrations (VIV) and Self-Excited Vibrations (SEV) are the two significant flow-induced vibrations that are exploited by piezoelectric energy harvesters [45,46].

To create VIV, a bluff body is located in a water stream, and depending on the characteristics of the bluff body, vortices of a particular size and frequency appear. If, then, the piezoelectric energy harvester is placed behind the body, the generated vortices make it oscillate. This oscillation will generate electricity based on the piezoelectric principle [45].

Through the use of self-excitation of flexible bodies, here most often PVDF materials, SEV-based energy harvesting can be achieved. The bodies are placed in the flow stream and, by a minute increase in flow speed, the body attains self-excitation, which can be further used for electrical energy generation [46].

#### 2.6.2. Wave Motion

Waves in the oceans are generated by the association of forces, such as wind, atmospheric pressure gradients, earthquakes, gravitational attraction, and storms. Moreover, these waves need to be restored using mechanisms such as surface tension, gravity, and Coriolis force. Based on their periods, waves can be classified into seven groups: (1) Capillary waves; (2) Ultra-gravity waves; (3) Gravity waves; (4) Infra-gravity waves; (5) Long-period waves; (6) Ordinary tidal waves; and (7) Trans-tidal waves [47].

**Figure 10:**
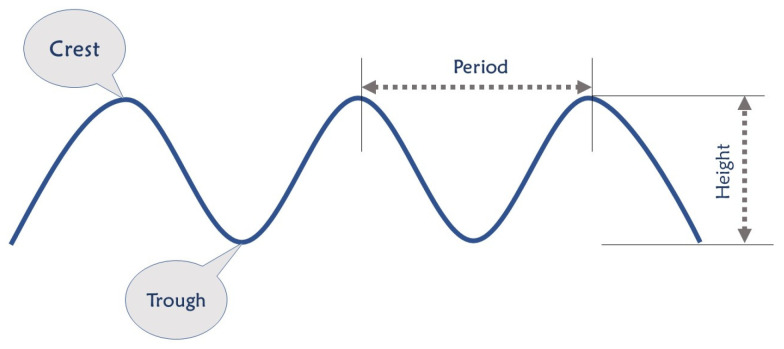
Characteristics of an Oceanic Wave.

There are two main factors in determining how much energy a wave holds (Figure 10): wave height and the wave’s period [48], meaning the time taken for a wave crest to travel the distance between two wave crests. Waves with a greater height and shorter periods contain more energy. However, unlike water currents where the energy is distributed all along, the waves’ energy density diminishes by the depth and is concentrated near the surface of the ocean. Therefore, the facilities’ tip that captures the wave’s energy should be close to the surface of the ocean [49]. According to [11,50], piezoelectric energy harvesters from wave motion mainly consist of heaving and pitching bodies, the PVDF layer on the surface of the ocean, and fixed bodies on the ocean bottom.

#### 2.6.3. Wave Impact

Another feature of the ocean is the impact of the waves on the ocean structure [50]. Mostly, wave impacts are considered destructive forces. However, these waves’ forces on the structures can be advantageous rather than being devastating. In the case that the piezoelectric energy harvesters are placed on the surfaces that are subjected to waves’ impact, they can convert the applied pressure to electricity [11].

### 2.7. Location

**Figure 11:**
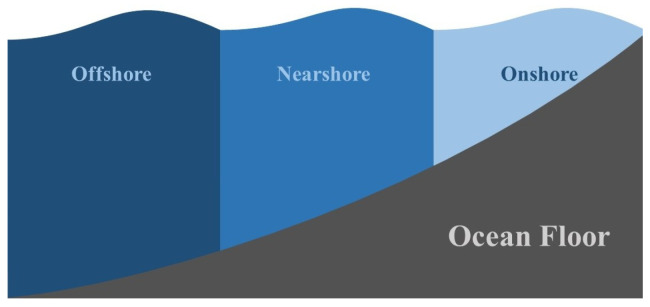
Ocean Regions.

Based on where the marine facilities are working, they can be categorized into three groups; onshore; nearshore; and offshore. Onshore represents regions with 10–15 m water depth, where this value is 15–25 m for nearshore, and is higher than 50 m for offshore. Figure 11 shows the different regions in the ocean [51].

## 3. The ATLAS

The following comprehensive atlas of 84 designs contains information on the utilized piezoelectric materials, piezoelectric coupling modes, location, power range, energy source, and a schematic of the piezoelectric energy harvesters design in oceanic engineering. It should be noted that all the information is collected exactly from the related references without any judgments and changes. The atlas includes just the information that the research articles provided, and in the cases where there is no information in any of the atlas’s sections, the related unit is free of information. Moreover, the atlas only includes designs for which they have at least provided a simulation or fabricated and tested a prototype. The schematics are sketched in a way to help future designers simply understand the working principles of the state-of-the-art. Table 1 shows the meaning of different colors in the schematics to fully understand the concepts. Table 2, Table 3, Table 4 and Table 5 show the atlases of cantilever beam-based, diaphragm-based, stacked-based, and cymbal-based designs, respectively.

## 4. Discussions and Research Needs

There are huge amounts of energy in oceans in different forms, which can be converted to electricity using many different technologies. Wave motion, water current, and wave impact are the three most commonly used sources of energy in piezoelectric energy harvesters to extract low-power electricity from the oceans. As completely reviewed, many methods have been developed to convert ocean energy using piezoelectric materials for low power devices, such as sensors and IoT things. There are, however, challenges in the field of piezoelectric energy harvesters in ocean engineering, which should be addressed for future developments. In order to develop the field, several directions are proposed to fill the gaps and enhance the performance of future designs.

### 4.1. Configurations

As discussed in Section 2.4, four different configurations are utilized to harvest electrical energy from the ocean by piezoelectric materials: cantilever beam; diaphragm; stacked; and cymbal configurations. Table 6 shows the range of output power in each of the aforementioned configurations for energy harvesting from oceans. As it is clear from Table 6, the cantilever beam-based configuration covers a wide range of output power in the oceanic application.

Ocean waves contain enormous energy inside but with a low frequency [134], which is the main challenge considering the high natural frequency of piezoelectric materials [11]. The cantilever beam configuration has a much lower resonance frequency compared to the other configurations. Moreover, by attaching a proof mass to the free end of the beam, its resonance frequency can be further lowered. Therefore, other than its simple structure and low cost, it can be easily employed for a higher range of output power, as well as various applications [34].

Introducing a medium device to translate low-frequency wave motion to a higher order of magnitude vibration is another solution for matching the resonance frequency of the oceanic waves with the energy harvester’s [56,126,127]. This solution opens a wide range of opportunities for the other configurations to be widely employed in oceanic applications. Being unable to withstand high impact forces makes cantilever beam-based configurations susceptible to damage by the high forces of the ocean waves [34]. Therefore, developing novel medium structures to match the resonance frequency of the other configurations with that of ocean waves seems necessary and can be considered as a gap in the development of the other configurations for a higher range of power outputs in oceanic applications [11].

As the waves’ energy density diminishes with depth and is concentrated near the surface of the ocean, it is of high importance to optimize the existing techniques and develop other novel strategies to excite piezoelectric materials by the low-frequency motion of the ocean waves to provide electricity for low-power devices at the surface of the ocean [11].

### 4.2. Material

**Figure 12:**
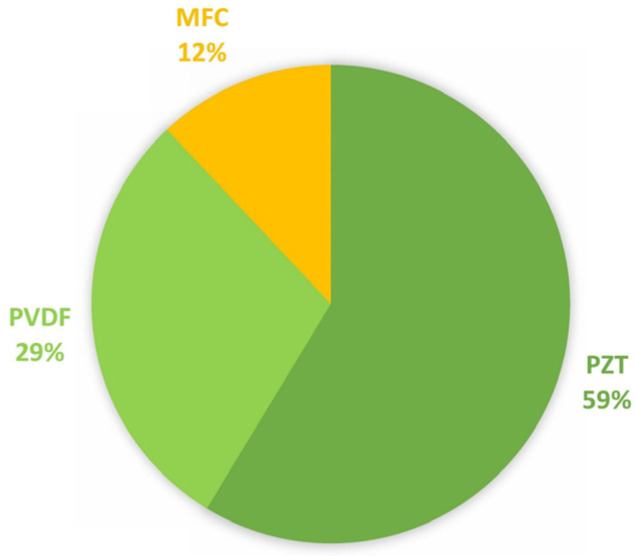
Frequency of different materials’ usage in oceanic applications.

According to Section 2.2, PZT, PVDF, and MFC are the main piezoelectric materials for harvesting electrical energy from the oceans. As shown in Figure 12, PZT is the most utilized material in ocean energy harvesting because of its remarkable properties (Table 7). In addition to excellent dielectric and piezoelectric properties and a higher d_31_ value, PZT also has a high Curie temperature, above which piezoelectric materials undergo a sharp change that leads to losing their piezoelectricity [135]. Over time, to make it applicable in diverse applications, its chemical compositions have been modified regularly, meaning that PZT maintains the largest family among piezoelectric materials with a wide range of material properties at a low cost [136]. Therefore, owning great properties at a low cost makes PZTs the first choice for energy harvesting applications. However, PZT is brittle and fragile by its nature; it shows an elongation of 0.1% at break. Therefore, PVDF, due to its flexibility, is utilized in applications that need to bear higher loadings. This material has a much lower Young’s modulus compared to that of PZT (Table 7) and shows an elongation of 10% at break. On the other hand, PVDF’s power density is much lower than that of PZT, which introduces issues related to the size of the harvester in applications where high output power is needed. MFC material seems to be a kind of trade-off between these two materials by having both higher flexibility and higher power density than PZT and PVDF, respectively [137]. It is highly recommended to consider MFCs in future designs for their great properties, such as a lower Young’s modulus than PZT and a higher d_31_ constant than PVDF.

It is also reported that the composites of PZT-PVDF show better performance in electrical and mechanical properties compared to MFCs [138]. This composite could be one of the most promising materials for future energy harvesters in oceanic applications. Therefore, it is highly suggested to consider PZT-PVDF composites in future designs of energy harvesters in oceanic applications.

### 4.3. Coupling Modes

**Figure 13:**
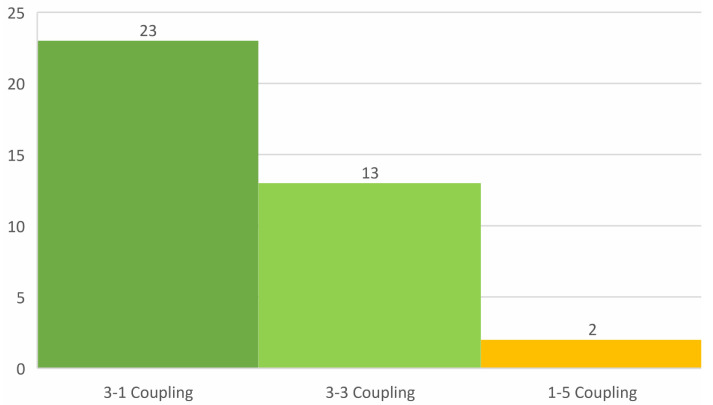
Frequency of different coupling modes’ usage in oceanic applications.

As completely reviewed in Section 2.3, according to [28], four different coupling modes are utilized in piezoelectric-based energy harvesters in oceanic applications: 3-1, 3-3, 1-4, and 1-5 modes. However, the 1-4 coupling mode is not directly mentioned in the recent research papers, and thus the developed atlas only contains 3-1, 3-3, and 1-5 coupling modes. Figure 13 illustrates the frequency of usage for different coupling modes for piezoelectric energy harvesters in oceanic applications. As can be seen in Figure 13, the frequency of 3-1 coupling usage is higher than the other two coupling modes. However, according to [26], the 3-1 coupling mode has the lowest piezoelectric coefficient and, consequently, it should have the lowest output power among these three coupling modes. The reason behind their wider applications is that the majority of the piezoelectric energy harvesters in oceanic applications are designed based on cantilever beams, and the 3-1 coupling mode is the best choice for a cantilever beam-based harvester since in this mode, the polar axis is not directly subjected to the input stress and applying large strains by bending deformation of the harvester perpendicular to the polar axis is much easier [26,135]. According to the atlas and [26,28], designs based on the 3-1 coupling mode are the simplest structures, such as a simple tape, a film, or a cantilever beam, which are bonded to a piezoelectric material for energy harvesting purposes. Although, in comparison to the 3-3 coupling mode, the 3-1 coupling mode produces a lower output voltage, it shows a larger output current [33].

Considering that the output power of the 3-1 coupling mode is 3 times and 3-6 times lower than that of the 3-3 and 1-5 coupling modes, respectively, it is of high significance to consider 3-3 and 1-5 coupling modes to improve the output power in future designs. Although employing a 1-5 coupling mode due to its shear configuration is challenging, it can lead to a higher power density in piezoelectric energy harvesters. Therefore, it is suggested to develop techniques to utilize 1-5 coupling modes in future designs for higher output powers.

### 4.4. Energy Sources

**Figure 14:**
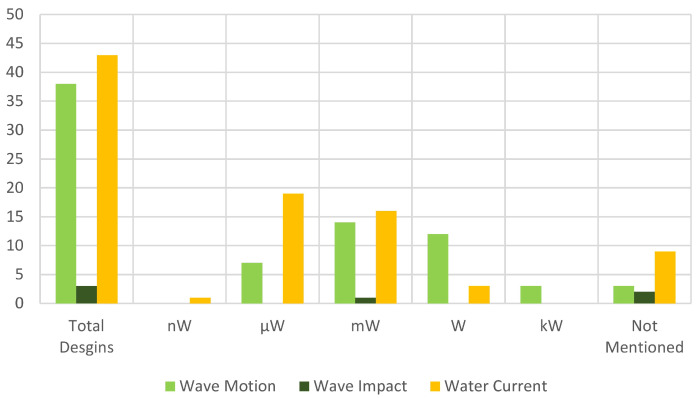
Comparison of the piezoelectric energy harvesters based on their energy sources and output power.

Section 2.6 discusses the different energy sources available in the oceans to convert energy from. Figure 14 summarizes the different designs based on the energy sources as well as their output power. Designs based on ocean wave motion show a higher output power compared to the other two energy sources. Energy harvesters based on ocean wave motion can be employed to power buoys and measurement devices, such as sensors. As discussed earlier, the main issue with ocean wave motion is their low frequency, which should be considered to be matched by that of the piezoelectric material [134]. On the other hand, energy harvesters based on water currents have an average power output applicable for underwater moving devices. Due to their low hydrodynamic efficiency, they can only utilize a small fraction of the water currents’ energy. To address this issue, many of these devices can be aligned to build a big farm to capture higher fractions of water currents’ energy [11,59,60]. The directions of the alignment need to be tuned carefully to reach the maximum available output power. The other concern with water current-based energy harvesters is their continuous contact with the ocean’s corrosive water, which should be considered in the design stage of these energy harvesters to select proper materials [11].

### 4.5. Power

**Figure 15:**
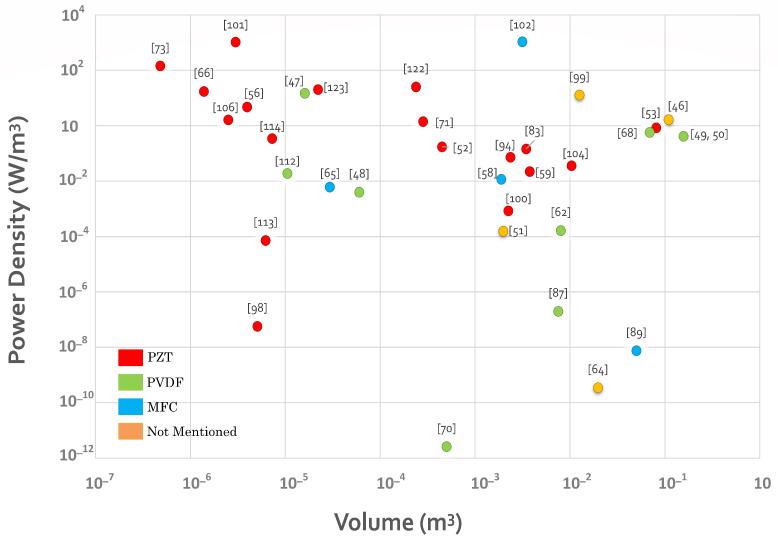
Power density per volume graph of the oceanic piezoelectric energy harvesters.

Piezoelectric energy harvesters provide many different application scenarios by having flexibility in their structures, coupling modes, sizes, etc. Therefore, the amount of generated energy is different from one harvester to the next. An identical and standardized criterion is needed to compare oceanic piezoelectric energy harvesters. Here, maximum power per volume (W/m^3^) is adopted to show the designed structures’ electrical performance. Moreover, as the volume of a harvester represents its structure, size, and applicability in different locations, it is used here to provide a comprehensive outlook on the state-of-the-art. Power density, as well as the volume of the designed piezoelectric energy harvesters, is utilized in Figure 15 to illustrate the relationship between designs in the literature. However, it is of high importance to mention that there may be errors/inaccuracies in Figure 15 due to: (1) power density and the volume of the designed devices may be computed in another way by the authors; (2) the number of designs that provided enough information for the Figure is limited; and (3) only the designs that have tested a prototype are considered for this analysis.

Several conclusions can be drawn based on Figure 15: (1) Among the three widely used materials in oceanic piezoelectric energy harvesters, PZT is used in a wide range of volumes and power densities. Due to its remarkable characteristics, it also shows the highest power density among the three materials used in oceanic applications; (2) MFC reaches the same high-power density as PZT but in a larger volume; and (3) As the piezoelectric coefficient of the PVDF is much lower than that of PZT and MFC, the volume of PVDF-based energy harvesters is higher than the others.

The statistics coming from Figure 13 can be used as a reference for material selection, harvester structure, power requirements, and the size of the energy harvester.

## 5. Conclusions

In this study, the main aspects of piezoelectric energy harvesters in oceanic applications are reviewed, and for more information, the reader is referred to the related references. Then, a comprehensive atlas of 85 piezoelectric energy harvesters, which are classified based on piezoelectric configurations (cantilever beam, diaphragm, stacked, and cymbal configurations), has been provided. The atlas includes information on material, piezoelectric coupling modes, location, power density, energy source, and finally, a unified schematic of the designs in the literature. Each design has been represented using its working principle, which is rather close to the original design. The collection includes designs that are fabricated or at least simulated. All the schematics are prepared in such a way that the working principle can be easily understood. In the end, the collection (atlas) has been analyzed in several aspects, such as configurations, materials, coupling modes, energy sources, and output power, to obtain research gaps and to help future designers in the field by making a recipe to select the right material, configuration, coupling mode, energy source, location, etc. The authors hope that this collection of designs will help researchers come up with their own energy harvesters for oceanic applications.

## Figures and Tables

**Table 1:** Meaning of each color in the atlas.

Color	Meaning
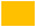	Buoy
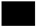	Magnet
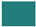	Piezoelectric Layer
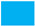	Water
The Other Colors	Non-Piezoelectric Material

**Table 2:** Atlas of cantilever beam-based piezoelectric energy harvesters.

Year and Reference	Material	Coupling Mode	Location	Power Density (W/m^3^)	Energy Source	Schematics
1987 [52]	PVDF	3-1	Onshore	-	Wave Motion	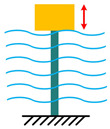
1987 [53]	PVDF	3-3	-	-	Wave Motion	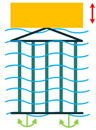
2001 [54]	PVDF	3-1	-	-	Water Current	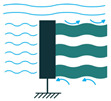
2004 [55]	PVDF	3-1	-	-	Water Current	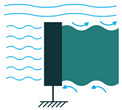
2004 [55]	PZT	3-1	-	70.00	Water Current	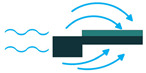
2007 [25]	PVDF	3-1	Onshore	-	Water Current	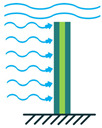
2009 [56]	-	-	Offshore	1.64	Wave Motion	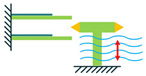
2010 [57]	PVDF	3-3	Offshore	15.00	Water Current	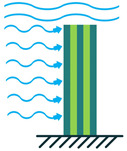
2011 [58]	PVDF	-	Offshore	4.00 × 10^−3^	Water Current	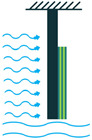
2012 [59,60]	PVDF	-	Offshore	0.42	Wave Motion- Water Current	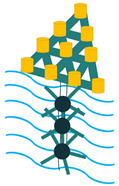
2012 [61]	-	-	-	1.56 × 10^−4^	Water Current	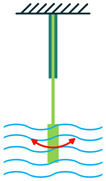
2012 [62]	PZT	3-1	Onshore	0.17	Wave Motion	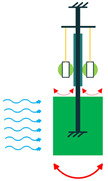
2013 [63]	PZT	3-3	-	0.84	Water Current	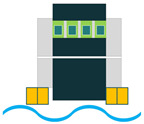
2013 [64]	PZT	-	-	366.00	Wave Motion	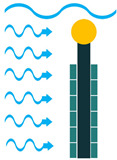
2014 [65]	PZT	-	Offshore	75.00	Wave Motion	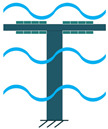
2014 [66]	PZT	3-3	-	4.74	Wave Motion	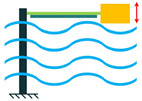
2014 [67]	PVDF	3-1	-	-	Water Current	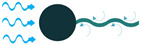
2015 [68]	MFC	3-1	-	1.16 × 10^−2^	Water Current	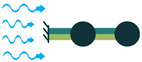
2015 [69]	PZT	3-1	-	2.24 × 10^−2^	Water Current	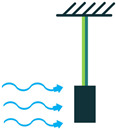
2015 [70]	PZT	-	-	-	Water Current	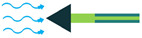
2015 [71]	PVDF	-	-	-	Water Current	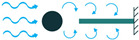
2015 [72]	PVDF	3-1	-	1.65 × 10^−4^	Water Current	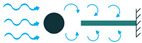
2015 [73]	PZT	-	Near/Offshore	1.27	Wave Motion	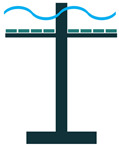
2015 [74]	-	-	Nearshore	3.50 × 10^−10^	Wave Motion	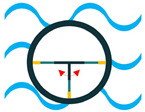
2015 [75]	MFC	-	-	6.10 × 10^−3^	Water Current	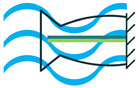
2016 [76]	PZT	3-1	-	17.31	Water Current	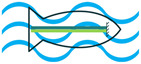
2016 [77]	PZT	3-3	Near/Offshore	206.00	Wave Motion	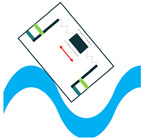
2016 [78]	PVDF	-	-	0.58	Water Current	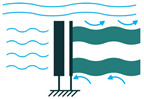
2016 [78]	PZT	-	-	0.58	Water Current	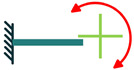
2016 [79]	PVDF	3-1	Near/Offshore	-	Wave Motion	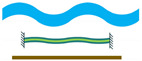
2017 [80]	PVDF	3-1	Near/Offshore	2.60 × 10^−12^	Water Current	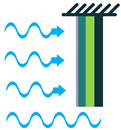
2017 [81]	PZT	-	-	1.40	Water Current	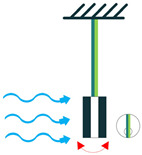
2017 [82]	PZT	-	-	1.59 × 10^4^	Wave Motion	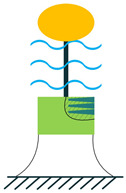
2017 [83]	PZT	-	-	143.54	Wave Motion	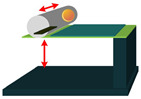
2017 [84]	PZT	3-3	On/Near/Offshore	260.00	Wave Motion	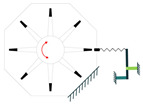
2017 [85,86]	PZT	3-1	-	3.50 × 10^−8^	Water Current	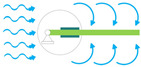
2018 [87]	PZT	3-1	-	-	Wave Motion	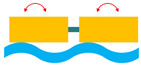
2018 [88]	PZT	-	Offshore	10.34 × 10^3^	Wave Motion	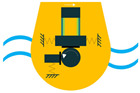
2018 [89]	-	3-3	-	2.40 × 10^3^	Wave Motion	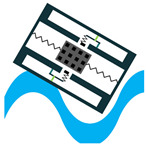
2018 [90]	MFC	3-1	-	-	Water Current	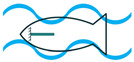
2018 [91]	-	-	-	2.56	Water Current	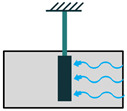
2019 [92]	-	-	-	-	Wave Motion	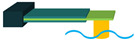
2019 [93]	PZT	3-3 & 1-5	-	0.14	Water Current	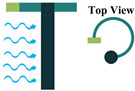
2019 [94]	PZT	3-3	Offshore	477.00	Wave Motion	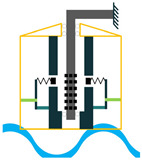
2019 [95]	PZT	3-1	-	-	-	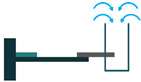
2019 [96]	PZT	-	Nearshore	1.06 × 10^4^	Wave Motion	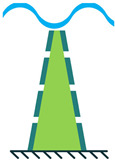
2019 [24]	-	3-1	Onshore	-	Wave Impact	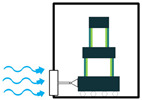
2019 [97]	PVDF	3-1	Offshore	2.00 × 10^−7^	Wave Motion	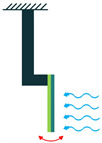
2019 [98]	-	-	-	-	Water Current	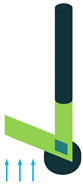
2019 [99]	MFC	-	-	7.58 × 10^−9^	Water Current	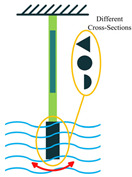
2019 [100]	MFC	-	-	-	Water Current	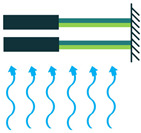
2019 [101]	PZT	-	-	-	Water Current	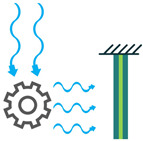
2019 [102]	PZT	-	Offshore	140.00	Wave Motion	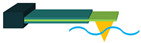
2020 [103]	PVDF	-	-	-	Water Current	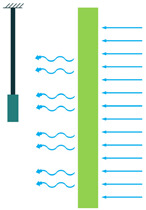
2020 [104]	PZT	3-1	-	7.34 × 10^−2^	Water Current	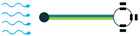
2020 [105]	MFC	-	-	-	Water Current	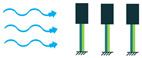
2020 [106]	PZT	-	-	-	Wave Motion	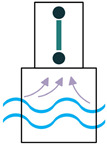
2020 [107]	PVDF	-	-	-	Water Current	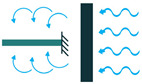
2020 [108]	PZT	-	-	5.74 × 10^−8^	Water Current	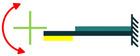
2021 [109]	-	-	-	12.90	Wave Motion	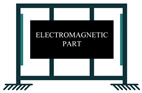
2021 [110]	PZT	-	-	8.42 × 10^−4^	Water Current	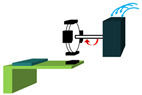
2021 [111]	PZT/PVDF	-	Near/Offshore	10.50 × 10^2^	Wave Motion	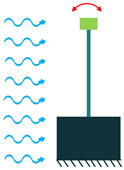
2021 [112]	MFC	3-1	-	10.74 × 10^2^	Water Current	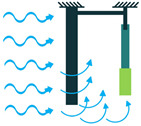
2021 [113]	PVDF	3-1	-	-	Wave Motion	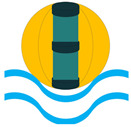
2021 [114]	PZT	-	-	3.58 × 10^−2^	Water Current	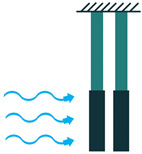
2021 [115]	-	-	-	3.20 × 10^−3^	Wave Motion	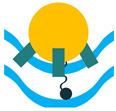
2021 [116]	PZT	-	-	1.63	Wave Motion	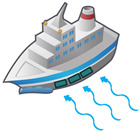
2021 [117]	-	-	-	-	Wave Motion	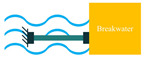
2021 [118]	PZT/MFC	-	-	62.00 × 10^3^	Wave Motion	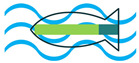
2021 [119]	PZT	-	Offshore	-	Wave Motion	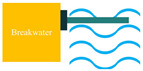
2021 [120]	PZT	-	-	-	Wave Motion	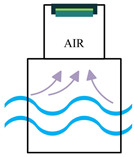
2021 [121]	-	-	Nearshore	-	Wave Motion	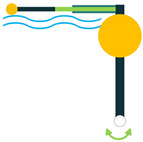
2021 [122]	MFC	3-3	-	8.50 × 10^−3^	Wave Motion	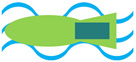

**Table 3:** Atlas of diaphragm-based piezoelectric energy harvesters.

Year and Reference	Material	Coupling Mode	Location	Power Density (W/m^3^)	Energy Source	Schematics
2010 [123]	PVDF	3-1	-	1.90 × 10^−2^	Water Current	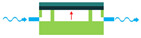
2011 [124]	PZT	1-5	-	7.25 × 10^−5^	Water Current	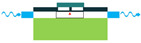
2013 [125]	PZT	-	Offshore	0.34	Water Current	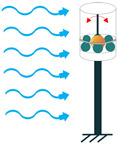
2013 [126]	PZT	-	Offshore	-	Wave Motion	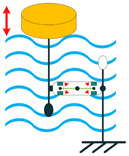
2013 [127]	PZT	-	Offshore	-	Wave Motion	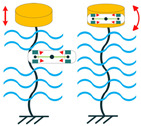
2018 [128]	PZT	3-3	-	-	Water Current	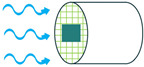
2019 [129]	PVDF	-	-	5.68 × 10^−2^	Wave Motion	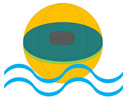

**Table 4:** Atlas of stacked-based piezoelectric energy harvesters.

Year and Reference	Material	Coupling Mode	Location	Power Density (W/m^3^)	Energy Source	Schematics
2013 [50]	PVDF	3-3	-	-	Wave Impact	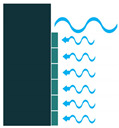
2017 [130]	PZT	-	Offshore	600.00	Wave Impact	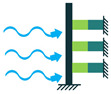
2021 [131]	PZT	-	-	-	Wave Motion	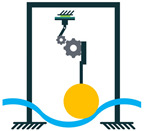

**Table 5:** Atlas of cymbal-based piezoelectric energy harvesters.

Year and Reference	Material	Coupling Mode	Location	Power Density (W/m^3^)	Energy Source	Schematics
2010 [132]	PZT	3-3	-	25.26	Water Current	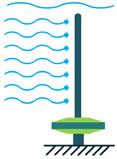
2021 [133]	PZT	-	-	20.38	Water Current	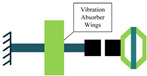

**Table 6:** Output power range in different configurations in the oceanic application.

Type of Configuration	nW	µW	mW	W	kW
Cantilever Beam	-	■	■	■	■
Diaphragm	■	■	■	-	-
Stacked	-	■	■	-	-
Cymbal	-	■	■	-	-

**Table 7:** Properties of piezoelectric materials used in oceanic energy harvesters.

Type of Material	Young’s Modulus (GPa)	d_31_ (×10^−12^ m/V)	k_31_	Dielectric Constant
PZT	62	320	0.44	~3800
PVDF	2–4	23	0.12	~12–13
MFC	16	170	-	-
